# Metagenomes in the Borderline Ecosystems of the Antarctic Cryptoendolithic Communities

**DOI:** 10.1128/MRA.01599-19

**Published:** 2020-03-05

**Authors:** Claudia Coleine, Davide Albanese, Silvano Onofri, Susannah G. Tringe, Christa Pennacchio, Claudio Donati, Jason E. Stajich, Laura Selbmann

**Affiliations:** a Department of Ecological and Biological Sciences, University of Tuscia, Viterbo, Italy; b Research and Innovation Centre, Fondazione Edmund Mach, San Michele all’Adige, Italy; c Department of Energy Joint Genome Institute, Walnut Creek, California, USA; d Department of Microbiology and Plant Pathology, University of California, Riverside, Riverside, California, USA; e Institute of Integrative Genome Biology, University of California, Riverside, Riverside, California, USA; f Mycological Section, Italian Antarctic National Museum, Genoa, Italy; Broad Institute

## Abstract

Antarctic cryptoendolithic communities are microbial ecosystems dwelling inside rocks of the Antarctic desert. We present the first 18 shotgun metagenomes from these communities to further characterize their composition, biodiversity, functionality, and adaptation. Future studies will integrate taxonomic and functional annotations to examine the pathways necessary for life to evolve in the extremes.

## ANNOUNCEMENT

Antarctic cryptoendolithic communities are microbial ecosystems dwelling inside airspaces of rocks under the superextreme conditions of the ice-free areas of continental Antarctica. In Antarctica, they were first described from the McMurdo Dry Valleys, which are considered one of the best analogues of the Martian environment on Earth and were thought to be devoid of life until the discovery of these cryptic life forms (1). The cryptoendolithic ecosystems are highly specialized, are adapted to exploit a narrow ecological niche, and represent excellent models to investigate how life can persist at the extremes of aridity, solar radiation, and temperature. Recent molecular and genomic studies have shed light on the structure and diversity of some biological functional groups found in these environments (2–7). Here, we report the first shotgun metagenomics of Antarctic endolithic communities to further examine the biodiversity, ecological functions, and potential stress response strategies of the community members. These genomic data can aid in developing models for the organisms from this extreme environment, including those to learn their identity, functional capabilities, ecosystem roles, and mechanisms of adaptation.

Eighteen colonized sandstones were aseptically collected, using a geological hammer and chisel, from sites in Victoria Land (continental Antarctica) along a latitudinal transect ranging from 74°10′44.0ʺS to 77°52′28.6ʺS, from 834 to 3,100 m above sea level, during the XXXI Italian Antarctic Expedition (2015 to 2016). Northern and southern sun-exposed rock surfaces at each site were sampled. Collected samples were immediately placed in sterile bags and were kept at −20°C throughout transport and storage at the University of Tuscia (Viterbo, Italy) until processing. Pieces of each sample were pulverized with a sterile hammer, and total DNA was extracted from 1 g of crushed rock using a PowerSoil kit (Mo Bio Laboratories, Carlsbad, CA, USA). DNA was used to prepare paired-end genomic libraries using Nextera DNA kits, at the U.S. Department of Energy (DOE) Joint Genome Institute (JGI), and was sequenced (2 × 151 cycles) on a NovaSeq system (Illumina, Inc., San Diego, CA).

The BBDuk v.38.25 tool was used to remove contaminants and to trim adapters and low-quality sequences. The procedure removed reads that contained ≥4 "N" bases, had an average quality score across the read of <3, or had a minimum length of ≤51 bp or 33% of the full read length. Trimmed, screened, paired-end reads were corrected using BFC v.r181 (8) (with parameters “-1 -s 10g -k 21”). Reads lacking mate pairs after trimming and quality control were also removed. The trimmed, corrected reads were assembled with metaSPAdes v.3.12.0 (9) (with parameters “-m 2000 --only-assembler -k 68 33,55,77,99,127 --meta”). Coverage was calculated by mapping the filtered sequence reads to the assembly using BBMap v.38.25 (https://github.com/BioInfoTools/BBMap) (with default parameters and “ambiguous=random”).

A total of 3,817,654,184 filtered reads were obtained after quality control, with a mean of 212,091,899 reads per sample (minimum, 135,633,610 reads; maximum, 266,930,788 reads), which were assembled into more than 10 million contigs across all samples, with a GC content of 58.4% ± 3.2% (mean ± standard deviation) and *N*_50_ of 37,096 ± 19,919 bp. The DOE JGI Metagenome Annotation Pipeline v.4.16.5 (10), part of the Integrated Microbial Genomes with Microbiome Samples (IMG/M) system v.4 (11), predicted a total of 21,647,468 protein-coding genes across all assemblies.

### Data availability.

The reads and assemblies were deposited under the NCBI accession numbers listed in Table 1. Assembly, gene prediction, and annotation data sets are available at the IMG/M website (https://img.jgi.doe.gov) and in the Zenodo repository (12).

**TABLE 1:** Accession numbers and data for the metagenomes in this study

SRA accession no.	BioProject no.	GenBank accession no.	IMG/M identification no.	*N*_50_ (bp)	Sampling site
SRP176584	PRJNA513364	JAABUH000000000	3300030517	18,453	Battleship Promontory, north
SRP176586	PRJNA513365	JAABUI000000000	3300031909	25,796	Battleship Promontory, south
SRP176587	PRJNA513366	JAABUJ000000000	3300031452	23,579	Mount New Zealand, north
SRP176592	PRJNA513367	JAABUK000000000	3300032162	30,100	Pudding Butte, north
SRP176590	PRJNA513368	JAABUL000000000	3300031453	45,310	Pudding Butte, south
SRP176595	PRJNA513369	JAABUM000000000	3300031451	22,828	Siegfried Peak, north
SRP176596	PRJNA513370	JAABUN000000000	3300031454	16,381	Siegfried Peak, south
SRP176600	PRJNA513372	JAABUO000000000	3300031449	35,176	Finger Mountain, south
SRP176598	PRJNA513373	JAABUP000000000	3300031460	15,853	Linnaeus Terrace, north
SRP176601	PRJNA513371	JAABUQ000000000	3300031520	45,912	Finger Mountain, north
SRP176608	PRJNA513375	JAABUR000000000	3300031450	81,671	University Valley, south
SRP176612	PRJNA513376	JAABUS000000000	3300031473	53,362	Trio Nunatak, north
SRP176609	PRJNA513359	JAABUT000000000	3300031447	18,466	Ricker Hills, north
SRP176611	PRJNA513360	JAABUU000000000	3300030523	16,877	Richard Nunatak, white sandstone
SRP176669	PRJNA513362	JAABUV000000000	3300031448	50,022	Knobhead, north
SRP176664	PRJNA513363	JAABUW000000000	3300031471	49,126	Knobhead, south
SRP176667	PRJNA513361	JAABUX00000000	3300031472	75,065	Richard Nunatak, red sandstone
SRP176606	PRJNA513374	JAABUY00000000	3300031470	43,747	University Valley, north
